# Identification and verification of HCAR3 and INSL5 as new potential therapeutic targets of colorectal cancer

**DOI:** 10.1186/s12957-021-02335-x

**Published:** 2021-08-21

**Authors:** Xuan Yang, Wangao Wei, Shisheng Tan, Linrui Guo, Song Qiao, Biao Yao, Zi Wang

**Affiliations:** 1 Guizhou University Medical College, Guiyang, 550025 Guizhou China; 2 Department of Oncology, Guizhou Provincial People’s Hospital, Guizhou 550002 Guiyang, China; 3 Tongren Municipal People’s Hospital, Guizhou 554300 Tongren, China

**Keywords:** Colorectal cancer, Bioinformatics, Biomarkers, HCAR3, INSL5

## Abstract

**Background:**

Colorectal cancer (CRC) is one of the most common cancers of the gastrointestinal tract and ranks third in cancer-related deaths worldwide. This study was conducted to identify novel biomarkers related to the pathogenesis of CRC based upon a bioinformatics analysis, and further verify the biomarkers in clinical tumor samples and CRC cell lines.

**Methods:**

A series of bioinformatics analyses were performed using datasets from NCBI-GEO and constructed a protein–protein interaction (PPI) network. This analysis enabled the identification of Hub genes, for which the mRNA expression and overall survival of CRC patients data distribution was explored in The Cancer Genome Atlas (TCGA) colon cancer and rectal cancer (COADREAD) database. Furthermore, the differential expression of HCAR3 and INLS5 was validated in clinical tumor samples by Real-time quantitative PCR analysis, western blotting analysis, and immunohistochemistry analysis. Finally, CRC cells over-expressing INSL5 were constructed and used for CCK8, cell cycle, and cell apoptosis validation assays in vitro.

**Results:**

A total of 286 differentially expressed genes (DEGs) were screened, including 64 genes with increased expression and 143 genes with decreased expression in 2 CRC database, from which 10 key genes were identified: *CXCL1*,* HCAR3*,* CXCL6*,* CXCL8*,* CXCL2*,* CXCL5*,* PPY*,* SST*,* INSL5*, and *NPY1R.* Among these genes, *HCAR3* and *INSL5* had not previously been explored and were further verified in vitro.

**Conclusions:**

HCAR3 expression was higher in CRC tissues and associated with better overall survival of CRC patients. INSL5 expression in normal tissue was higher than that in tumor tissue and its high expression was associated with a better prognosis for CRC. The overexpression of INSL5 significantly inhibited the proliferation and promoted the shearing of PARP of CRC cells**.** This integrated bioinformatics study presented 10 key hub genes associated with CRC. *HCAR3* and *INSL5* were expressed in tumor tissue and these were associated with poor survival and warrant further studies as potential therapeutic targets.

**Supplementary Information:**

The online version contains supplementary material available at 10.1186/s12957-021-02335-x.

## Introduction

Colorectal cancer (CRC) incidence and mortality have been consistently increasing year-over-year. The 2020 GLOBOCAN statistics show that the incidence of CRC makes it the third most common form of cancer worldwide and it has the second highest mortality rate [1]. The prognosis of CRC is much better if diagnosed at earlier stages. Based upon aggregate data, a statistical analysis showed that the 5-year survival rate was over 90% for patients with stage I CRC compared to about 10% for patients with stage IV CRC [2]. Treatments for CRC include surgery, peri-operative radiotherapy plus chemotherapy, targeted therapy, and immunotherapy. Because of advancements in understanding colorectal carcinogenesis, the array of treatment options for local and advanced CRC have increased and individual treatment plans have also been developed [3]. The average survival time of advanced CRC has significantly improved in the past decade [4, 5]. However, survival is still poor for those with metastasized CRC. Thus, a further understanding of CRC pathogenesis and the identification of more prognostic markers are required.

Bioinformatics, one of the newest interdisciplinary fields incorporating biological research and computer science, has been widely used to exploring genetic correlations in tumors [6]. Recent studies have focused upon identifying core genes involved in pathogenesis and effective candidates for targeted therapy of CRC have been selected. Wang et al. identified a total of 202 differentially expressed genes (DEGs), including 58 genes with increased expression and 144 with decreased expression in CRC samples compared to normal tissues. A total of 10 hub genes were identified by cyto-Hubba: *PYY*,* CXCL3*,* CXCL11*,* CXCL8*,* CXCL12*,* CCL20*,* MMP3*,* P2RY14*,* NPY1R*, and *CXCL1* [7]. Xu et al. found a total of 87 DEGs, including 19 genes with increased expression and 68 with decreased expression, and showed that *SST*, *CXCL8*, and *MS4A12* were significantly differentially expressed between colorectal cancer tissues and normal tissues validated with RT-PCR assays [8]. Zhou et al. indicated that three novel genes: *CNTN3*,* SLC1A1*, and *SLC16A9*, which had diagnostic values with respect to occurrences of colorectal cancer, from a total of 237 identified differentially expressed genes [9]. Some CRC-related key mRNAs were also identified that increased understanding of molecular mechanisms underlying CRC development [10]. A bioinformatics approach has also been used in colonic adenoma, CRC metastases, and normal colonic epithelium samples. A total of 438 genes were identified to be differentially expressed in colonic adenoma, 885 in carcinoma, and 736 in metastases [11].

However, these biomarkers have not been sufficiently explored for potential clinical applications; largely because the aforementioned studies did not verify the identified genes in cell lines or CRC samples using molecular biological techniques. Accordingly, this study was conducted to identify and verify biomarkers using a novel data combination analysis for diagnosing and exploring the pathogenesis for new CRC therapeutic targets, and further verify them in clinical tumor samples and CRC cell lines.

## Materials and methods

### Patients and specimens

We retrospectively collected CRC tissues, adjacent non-tumor tissues, and paraffin-embedded specimens (except for the sample 2 missed the paraffin-embedded specimens) from 5 CRC patients (including 3 males and 2 females) under-going curative resection at the Tongren Municipal People’s Hospital from June 2020. Patients did not receive any preoperative anticancer treatment, including chemotherapy or radiotherapy. The mean age of these patients at the time of diagnosis was 66.8 ± 11.1 (range 46–77 years). The use of human samples for this project was approved by the ethics committee of Tongren Municipal People’s Hospital and written informed consent was obtained from each patient.

### Microarray data

The gene expression profiles from GSE9348 and GSE11024 were downloaded from GEO (https://www.ncbi.nlm.nih.gov/geo/) database. The GSE9348 data was generated using GPL570, [HG-U133_Plus_2] Affymetrix Human Genome U133 Plus 2 Array. Tumors from age and ethnicity matched 70 patients and biopsies from 12 healthy controls were used. The GSE110224 data was generated using GPL570, [HG-U133_Plus_2] Affymetrix Human Genome U133 Plus 2 Array, which consists of 17 normal colon tissue specimens and 17 tumor colon tissue specimens. The R software package was used to process the downloaded files and to convert and reject the unqualified data. The data was calibrated, standardized, and log2 transformed.

### Screening for DEGs

The ID corresponding to the probe name was converted into an international standard name for genes (gene symbol). The differential gene expression analysis was performed using the limma package in the Bio-conductor package (http://www.bioconductor.org/). The DEGs in colorectal cancer and normal colorectal samples from two microarray datasets were considered to have a corrected *P* value of < 0.05 and | log2 fold change (FC) > 2|. Additional graphics packages were produced to visualize the number of differentially expressed genes (DEGs) and heatmaps. A list of genes that an increased or decreased expression in both the microarray datasets was used for the subsequent analyses.

### Functional enrichment analysis

The enrichment analysis for Gene Ontology (GO) terms and Kyoto Encyclopedia of Genes and Genomes (KEGG) pathways was conducted using the R/Bioconductor package Cluster Profiler (http://www.bioconductor.org/packages/release/bioc/html/clusterProfiler.html) package, which was also used to plot the data. Only the GO terms and pathways with an adjusted *p* value < 0.05 were considered statistically significant..

### PPI network integration

To generate an interaction network, we uploaded common different gene identifiers to the STRING database (http://string-db.org/). The STRING database is a software system that is commonly used to identify the interactions between known/predicted proteins. Additionally, Cytoscape (www.cytoscape.org/) software was used to further expand upon the obtained interaction network. Each node is a gene, protein, or molecule, and the connections between the nodes represent the interactions between the molecules. This network was used to identify interactions and pathway relationships between the proteins encoded by the DEGs in CRC. The corresponding proteins in the central node are possible core proteins or key candidate genes with potentially important regulatory or physiologically relevant functions.

### The expression levels and survival analysis of Hub Genes in CRC

The mRNA expression of the hub genes and the overall survival of CRC patients were examined by data mining using The Cancer Genome Atlas (TCGA) Colon and Rectal Cancer (COADREAD) using the University of California, Santa Cruz (UCSC) Xena browser (https://ucscxenabrowser.net). The gene expression analysis was performed together with the computation of the associated box plots in R using the ggstatsplot (https://indrajeetpatil.github.io/ggstatsplot/). Survival curves were plotted using the Kaplan–Meier method and compared using log-rank test by R “survival” (https://github.com/therneau/survival) package. *P* values of < 0.05 were considered statistically significant.

### Cell culture

CRC cell lines (Stem Cell Bank, Chinese Academy of Sciences, CN) were maintained in McCoy’s 5A Medium (Gibco, USA) with 10% fetal bovine serum (NATOCOR, AR), 100 U/ml penicillin and 100 U/ml streptomycin. All cultures were maintained at 37 °C in a humidified atmosphere of 95% air and 5% CO_2_. *INSL5* cDNA was subcloned into the MSCV-IRES-mCherry-T2A-Puro retroviral vector. The retrovirus were produced using calcium phosphate-mediated transfection to produce stably transduced cells, which were then selected with 1 µg/ml puromycin (Meilun, CN) prior to sorting for mCherry fluorescent protein expression.

### CCK-8 assay

A CCK-8 assay kit (Targetmol, USA) was used to measure the proliferation of HT29 and SW620 cells. A total of 5000 cells in a volume of 100 μL per well in five replicate wells were cultured in a 96-well plate in medium containing 10% FBS. Then, the CCK-8 reagent (10 μL) was added to McCoy’s 5A Medium to generate a working solution, for which 100 μL was added per well and incubated for 2 h. A microplate reader was used to detect the absorbency at a wavelength of 450 nm.

### Cell cycle

Cells were treated with 20 μM BrdU (Sigma, DE) 48 h after seeding; 2 h later, the cells were harvested by centrifugation, followed by fixing with 0.4% formaldehyde for 20 min and washing with PBS. The cells were then fixed in 70% ethanol at – 20 °C for 12 h. The cells were permeabilized with 2 N HCL/0.5% Triton X-100 for 20 min and neutralized with 0.1 M sodium borate for 5 min prior to incubation with the anti-BrdU antibody (BD Biosciences, USA) for 20 min. Finally, the cells were resuspended in 500 µl with 10 µg RNase A for 30 min. The samples were counterstained with 2 µl Propidium Iodide (PI) for 15 min. The cell cycle distribution was analyzed by CytoFLEX S (Beckman, USA) and analyzed with the Flow Jo 10.0.5 (BD, USA).

### Cell apoptosis

Based upon the results of the CCK-8 assay, the SW620 cells were used for the Annexin V-FITC/PI cell apoptosis assay (BD Biosciences, USA). SW620 cells were seeded in 6-well plates at a density of 2 × 10^5^ cells/well. After 48 h, the cells were harvested, washed with PBS, and stained with the Annexin V/(PI) Apoptosis Detection Kit. The fluorescence of the cells was visualized using flow cytometry, for which the simultaneous detection of apoptosis pathway related protein levels was conducted.

### Real-time quantitative PCR (RT-PCR) analysis

Total RNA was extracted from patient tissue/cells with TRIzol Reagent (Invitrogen, USA). The total cellular RNA was reverse transcribed using an *Evo M-MLV RT* Kit with a gDNA Clean for qPCR kit (Accurate, CN). Blank reactions with no RNA were performed for all the experiments. The mRNA expression was measured by real-time PCR using SYBR Green Premix *Pro Taq* HS qPCR Kit (Accurate, CN). The CFX Connect TM Real-Time System (Bio-Rad, CA, USA) was used to run the RT-PCR reactions. The primer sets were as shown in Table S1. The relative gene expression to β-actin was calculated using the 2^−ΔΔCT^ method.

### Western blotting analysis

The protein levels for the molecules of interest were determined by western blotting according to the manufacturer’s protocol. The following antibodies were used: rabbit anti-HM74 (ER63689, HuaAn, CN), rabbit anti-INSL5 (ER60301, HuaAn, CN), rabbit anti-Bcl-2 (ET1702, HuaAn, CN), mouse anti-P21 (RT1449, HuaAn, CN), rabbit anti-Cleaved PARP (5625S, Cell signaling, USA), rabbit anti-P53 (2524S, Cell signaling, USA), rabbit anti-BAX (50,599, Cell signaling, USA), rabbit anti-Caspase-3 (A0214, ABclonal, CN), mouse anti-β-actin (M1210, HuaAn, CN), and anti-GAPDH-HRP (ET1702-66, HuaAn, CN). β-actin and GAPDH were used for the protein loading controls and analyzed with the Invitrogen iBright CL1500 Imaging System (Thermo Fisher Scientific, MA, USA). All determinations were repeated in triplicate.

### Histological characterization

Tumor samples and adjacent normal tissues from patients were fixed in 10% neutral buffered formalin. Paraffin blocks were prepared and serial 5-μm-thick sections were cut from paraffin-embedded tumors by a Paraffin cutter (Leica, DE). The paraffin sections were deparaffinized and rehydrated for subsequent immunohistochemical staining against HCAR3 and INSL5. Following antigen retrieval, endogenous biotin activity was blocked using normal bovine serum and the sections were incubated with primary antibody in a humidified chamber. Horseradish peroxidase-conjugated secondary antibody was applied to the sections, followed by incubation with DAB (3,3′-diamino-benzidine) substrate for color development. The expression levels of the molecules were estimated by optical microscopy (OLMPUS, JP) and the images were captured using a Leica micro-imaging system (Lecia, DE).

### Statistical analysis

All the experiments were performed independently in triplicate. These data points are presented using the mean ± the standard errors of the mean (SEM). Statistical significance of experimental data was determined by a non-parametric test. Statistical analyses were performed using GraphPad Prism 8 (GraphPad Software Inc., USA). For all tests, significant differences were indicated using *P =  < 0.05, **P < 0.01, or ***P < 0.001.

## Results

### Microarray data characteristics and the identification of DEGs in CRC

**Fig. 1:**
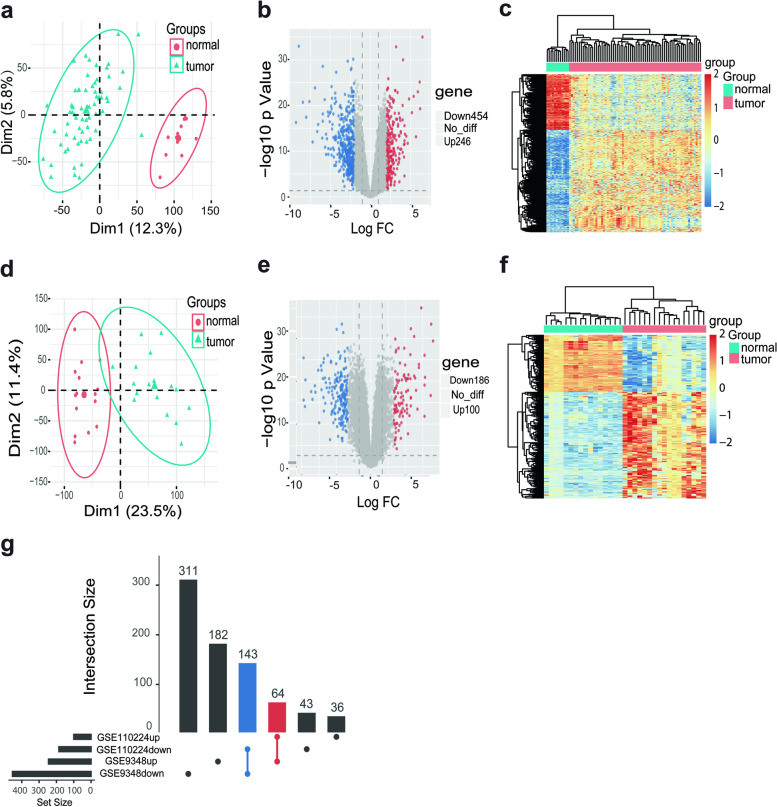
Differential expression of data between two sets of samples. Principal component analysis plot of gene expression data showing the first two principal components in GSE9348 (**a**) and GSE11024 (**d**). Volcano plot of the differential expression of genes in GSE9348 (**b**). Principal component analysis plot of the gene expression data showing the first two principal components in GSE11024 (**e**), respectively. Hierarchical clustering heatmap for GSE9348 (**c**) and GSE11024 (**f**), DEGs screened on the basis of a fold change > 2.0 and a corrected *P* value of < 0.05. (**g**) Upsetplot showing the number of DEGs in CRC tissues compared to that of normal colorectal tissues

The CRC expression microarray datasets GSE9348 and GSE110224 were calibrated and standardized, and the results are shown in Fig. 1a, d. When dataset GSE9348 was screened with the limma package (to identify genes with a corrected *P* value < 0.05, log FC > 2), 700 DEGs were obtained. Among them, 454 genes with decreased expression and 246 genes with increased expression were identified. Overall, 286 DEGs were identified in the GSE110224 dataset, including 100 genes with increased expression and 186 genes with decreased expression. The differential expression of genes from the two sets of sample data included in each of the two microarrays are shown in Fig. 1b, e. The cluster heatmaps for the DEGs are shown in Fig. 1c, f. Further analysis found that the two independent datasets contained 207 common DEGs, including 64 genes that had increased expression and 143 with decreased expression in both the CRC datasets (Fig. 1g).

### GO and KEGG pathway enrichment analysis

**Fig. 2:**
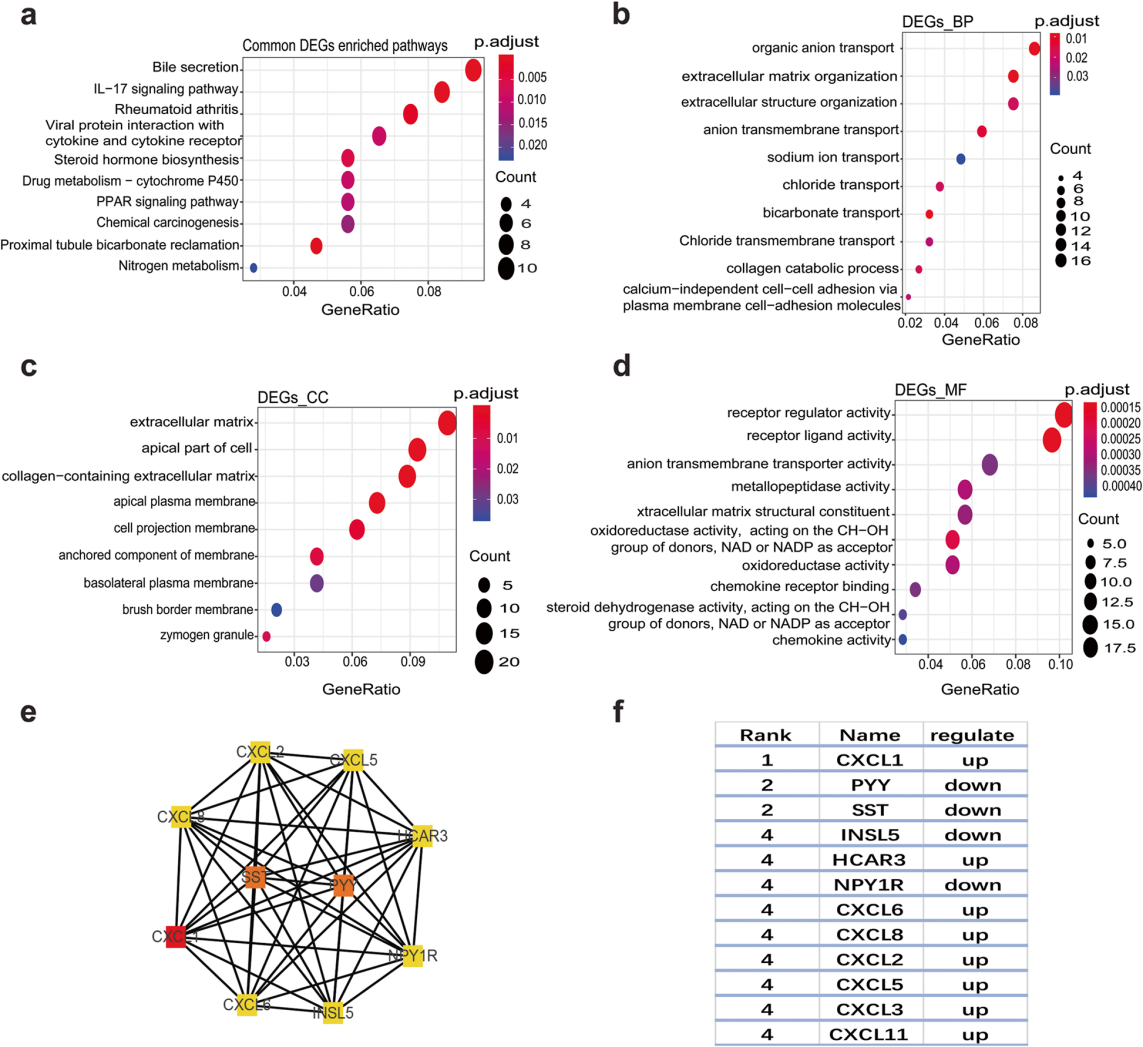
Integrated analysis of the common DEGs and the Protein–protein interaction (PPI) network.** a** KEGG pathways. **b** GO terms for biological processes. **c** GO terms for cellular components. **d** GO terms for molecular functions. **e** PPI network. **f** Hub genes

The common DEGs identified in the CRC datasets were further analyzed using a GO term and KEGG pathway enrichment analysis. The results of the KEGG enrichment pathway analysis showed that the signaling pathways enriched among the DEGs, included: bile secretion, IL-17 signaling pathway, rheumatoid arthritis, and PPAR signaling pathways (Fig. 2a). To understand the molecular basis, the DEGS were further categorized into molecular functions (MF), cellular component (CC), and biological process (BP) with a GO enrichment analysis. For the BPs, the common significantly enriched DEGs included organic anion transport and extracellular matrix organization (Fig. 2b). For CC, the common significantly enriched DEGs included extracellular matrix, apical part of cell, and collagen-containing extracellular matrix (Fig. 2c). For MF, the common enriched DEGs included receptor regulator activity, receptor ligand activity, and anion transmembrane transporter activity (Fig. 2d). Overall, these results indicated that the DEGs were mainly involved in extracellular matrix, extracellular matrix organization, organic anion transport, and receptor regulator activity. These are consistent with previous reports [12].

### Analyzing DEGs in colorectal cancer using a PPI network

The aforementioned (207) colorectal cancer DEGs were analyzed using the STRING database to construct PPI networks. The data was imported into Cytoscape to calculate the topological characteristics of the network and determine each node. Six genes with increased expression and four genes with deceased expression in were selected as the hub genes, including *CXCL1*, *HCAR3*, *CXCL6*, *CXCL8*, *CXCL2*, *CXCL5*, *PPY*, *SST*, *INSL5*, and *NPY1R* (Fig. 2e, f). An additional network analysis was conducted to assess the interactions of some of the hub genes with selected competing endogenous RNAs (Figure S1).

A comprehensive literature search was carefully conducted and two novel core genes, *HCAR3* and *INSL5*, had never previously been reported as being associated with CRC pathogenesis and progression.

### HCAR3 was downregulated in CRC associated with poor survival

**Fig. 3:**
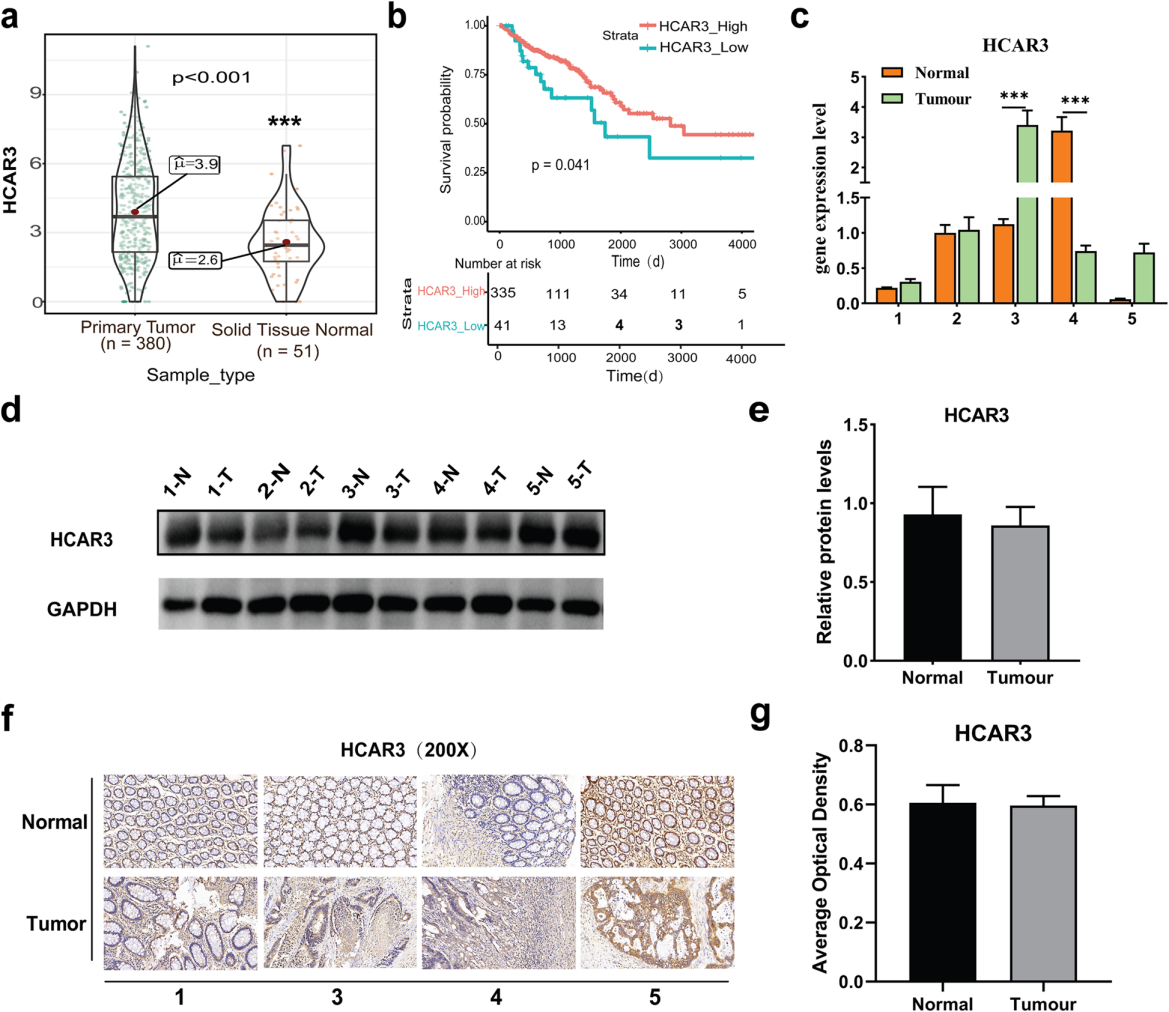
The expression of HCAR3 in human colorectal cancer. **a***HCAR3* mRNA expression was significantly higher in colorectal cancer than in normal colorectal tissue samples (***, *P* < 0.001). **b** Relationship between *HCAR3* expression level and prognosis of patients with colorectal cancer (*, *P* = 0.041). HCAR3 expression was detected in tumor tissues and adjacent normal tissues by RT-PCR (**c**), WB (**d**, **e**), and IHC (**f**, **g**). *, *P* =  < 0.05; **, *P* =  < 0.01; ***, *P* =  < 0.001

HCAR3 is a receptor for 3-OH-octanoid acid and a low affinity receptor for nicotinic acid, which mediates the negative feedback regulation of adipocyte lipolysis [13]. Previous studies reported that higher expression of *HCAR3* was negatively correlated with survival time of cervical cancer patients [14]. In CRC patients, the average expression of *HCAR3* was increased in CRC tissues compared to normal tissues (Fig. 3a). The expression of *HCAR3* was positively correlated to survival time of CRC patients (Fig. 3b). *HCAR3* expression changes were also detected with qPCR in five CRC patient samples. The RT-PCR results showed that in patients 1, 3, and 5, *HCAR3* was significantly increased in CRC tissues; in patient 2, there was no significant change between CRC tissue and normal tissue; whereas for patient 4, *HCAR3* was significantly decreased in CRC tissues compared to the normal tissue (Fig. 3c). The protein level of HCAR3 in these 5 CRC samples was also evaluated using western blotting. However, there were no obvious protein level changes for any of the samples (Fig. 3d, e). IHC assays were also performed for these samples, which were consistent with western blot results, and there was no significant HCAR3 protein signal increase compared to the adjacent non-tumor tissues (Fig. 3f, g).

### High expression of INSL5 was associated with a better prognosis

**Fig. 4:**
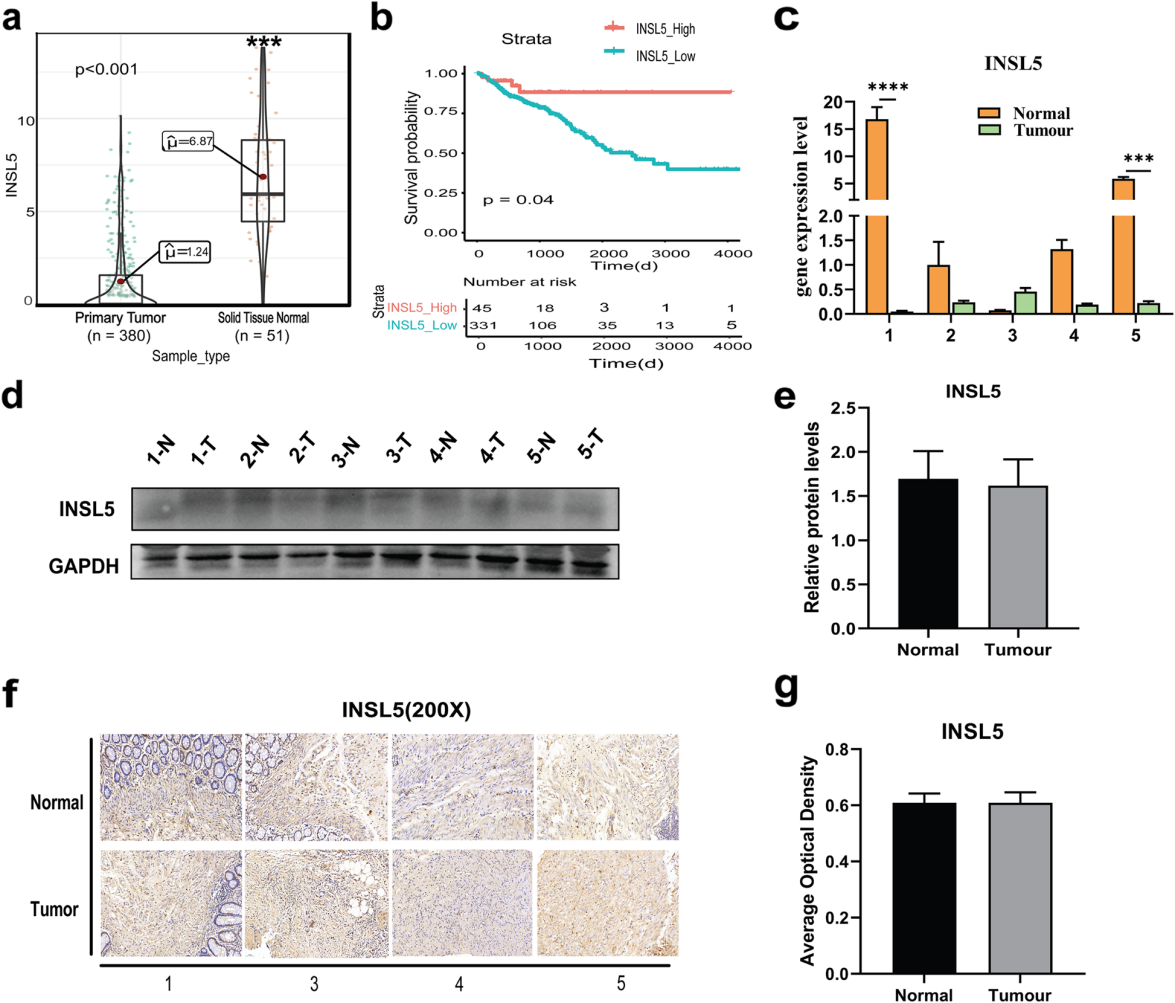
The expression of INSL5 in human colorectal cancer.** a** Expression of *INSL5* is reduced in colorectal cancer (***, *P* < 0.001). **b** The association of high-*INSL5* expression with a good prognosis is highly statistically significant (*, *P* = 0.04). INSL5 expression was detected in tumor tissues and adjacent normal tissues by RT-PCR (**c**), WB (**d**, **e**), and IHC (**f**, **g**). *, *P* =  < 0.05; **, *P* =  < 0.01; ***, *P* =  < 0.001

Based upon previous reports, higher expression of *INSL5* promoted nasopharyngeal carcinoma (NPC) progression [15]. INSL5 belongs to the insulin superfamily [16] and may play a role in gut contractility and thymic development/regulation [17]. The expression of *INSL5* was decreased in CRC tissue compared to normal tissue (Fig. 4a), and the expression of *INSL5* was associated with a better prognosis for CRC patients (Fig. 4b). *INSL5* expression status in CRC patient tissues was assayed with RT-PCR. *INSL5* expression was significantly reduced in 4 samples, but not patient 3 (Fig. 4c). The protein level of INSL5 in these 5 CRC samples was also evaluated with western blotting. No obvious changes were detected for the INSL5 protein bands (Fig. 4d, e). IHC assays were also performed to show the endogenous INSL5 level in CRC tissues, but no obvious protein signal changes were observed for all the detected samples (Fig. 4f, g).

**Fig. 5:**
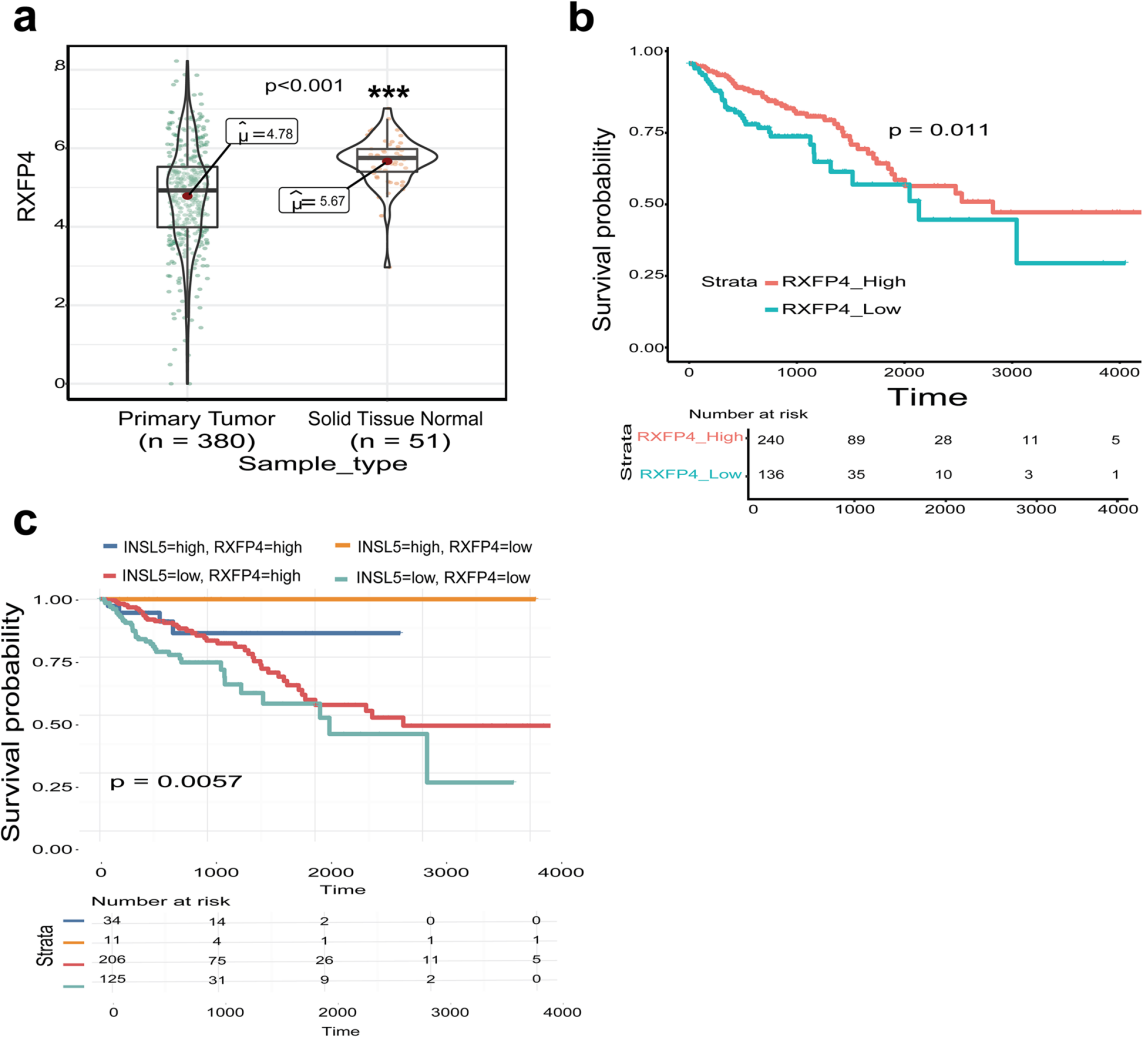
The expression of RXFP4 in human colorectal cancer and Kaplan–Meier survival curve analysis for INSL5/RXFP4 expression in colorectal cancer.** a** Expression of *RXFP4* is reduced in colorectal cancer (***, *P* < 0.001). **b** High *RXFP4* expression has better prognosis compared with low-*RXFP4* group (*P* = 0.011). **c** Kaplan–Meier survival curve of *INSL5/RXFP4* expression in colorectal cancer patients

**Fig. 6:**
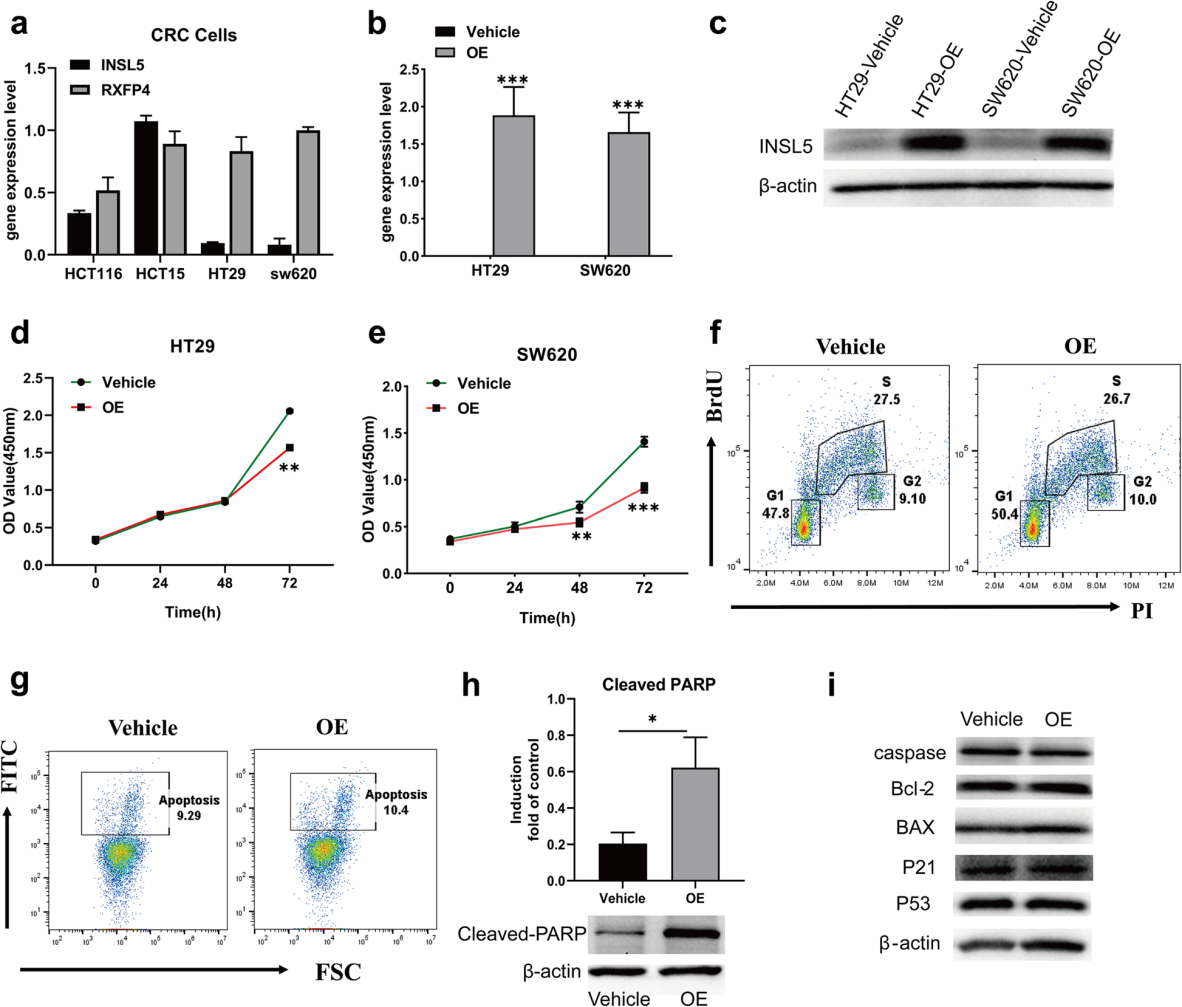
Over-expression of INSL5 inhibited tumor cell proliferation.** a** Expression of *INSL5* and *RXFP4* in CRC cells. **b**, **c** Stable overexpression for INSL5 by retroviral transfection in HT29 and SW620 cells. **d**, **e** Results of CCK-8 assays indicated that INSL5 significantly inhibited proliferation. **f** Results of cell cycle in SW620. **g**–**i** Results of cell apoptosis in SW620. The experiments were repeated thrice and similar results were obtained. Representative data was shown. Each assay was performed at least in triplicate. *, *P* =  < 0.05; **, *P* =  < 0.01; ***, *P* =  < 0.001

INSL5 endogenous receptor is RXFP4, which belongs to GPCR family, also named GPCR142 [18]. INSL5 binding to RXFP4 results in inhibited cAMP accumulation [19]. Consistently, both *INSL5* and *RXFP4* are specifically and highly expressed in gastrointestinal tract. Therefore, the expression of *RXFP4* in CRC tissue was also analyzed. Whilst the expression of *RXFP4* was significantly lower in the tumor tissues (*p* < 0.001), the *RXFP4* mRNA level was still maintained at a high level (Fig. 5a). The relationship between CRC prognosis and *RXFP4* expression was also analyzed in TCGA database, where higher expression of *RXFP4* was associated with better prognosis (Fig. 5b). The correlation between *INSL5* and *RXFP4* expression in CRC patients was determined to indicate whether the two genes were functionally relevant to prognosis. The best prognosis was observed for patients with high *INSL5*, as opposed to high expression levels of both *INSL5* and *RXFP4* (Fig. 5c). To further model this change, the expression of *INSL5* and *RXFP4* was determined in the CRC cell lines, of which HT29 and SW620 cells were selected to construct a stable overexpression system for INSL5 (Fig. 6a–c). CCK-8 assays demonstrated that overexpression of INSL5 significantly inhibited the proliferation of HT29 and SW620 cells (Fig. 6d, e). The result of cell cycle analysis found that overexpression of INSL5 did not significantly affect the cell cycle (Fig. 6f). Since the cells had the same mCherry fluorescence and PI detection channels, only FITC was used to indicate the total apoptosis of the cells (Fig. 6g). At the same time, the expression of the following apoptosis-related proteins was detected: Caspase 3, Cleaved-PARP, Bcl-2, BAX, P53 and P21. Except for Cleaved-PARP, the levels of the other proteins were not obviously different. Overexpression of INSL5 promoted the expression of Cleaved-PARP, which suggests that overexpression of INSL5 could promote the shearing of PARP (Fig. 6h, i).

## Discussion

In this study, an integrated bioinformatics analysis was performed based upon the gene expression profiles of GSE9348 and GSE11024, which were obtained from the GEO database. The DEGs in CRC were analyzed using a GO term and KEGG signal pathway enrichment analysis. The major significantly enriched annotations were functions or pathways involved in extracellular matrix, extracellular matrix organization, organic anion transport, apical part of cell, and receptor regulator activity (Fig. 2). This analysis enabled the identification of 10 hub genes by constructing a PPI network analysis. Most of these had been reported in previous studies. Among these studies, the authors reported that *CXCL1* and *CXCL5* played roles in CRC progression and metastasis, and high expression levels of *CXCL1* and *CXCL5* were associated with poor prognosis in patients with CRC [20, 21]. The CXC chemokine family appeared to have a complicated and multifaceted involvement in CRC. Tseng and Liu [22] have reported that lower expression of *PYY* may relate to the development and progression of CRC. SST is one of the cyclic tetradecapeptide hormones related to cancer growth, invasion, and metastasis [23]. A correlation between NPY1R and intestinal inflammation had previously been made, and *NPY1R* signaling was speculated to be a potential therapeutic target for colonic inflammation [24]. Two of the 10 hub genes, *HCAR3* and *INSL5*, had not previously been found in prior studies [20, 21].

*INSL5* is a member of the human insulin superfamily with an insulinotropic effect in colonic enteroendocrine L cells. As the endogenous ligand of RXFP4, INSL5 activates RXFP4 with high potency [25]. Mashima et al. [16] previously reported that INSL5 might be a unique marker of colorectal EECs and INSL5–RXFP4 signaling might play a role in an autocrine/paracrine fashion in colorectal epithelium and rectal neuroendocrine tumors. A recent study reported that *INSL5* enhanced NPC progression and indicated that *INSL5* might be a therapeutic target for NPC [15]. However, the study presented herein revealed that high expression of both *INSL5* and *RXFP4* was associated with a better prognosis for CRC (Fig. 4b and Fig. 5). However, the robust expression of RXFP4 in CRC indicated that its biological activity may not be greatly impaired. The differential expression of *RXFP4* mRNA between the tumor and normal tissue may not be the major factor for CRC prognosis. This hypothesis was confirmed when CRC prognosis was further analyzed, which accounted for both *INSL5* and *RXFP4* expression. Indeed, the expression of *INSL5* was the decisive factor for CRC prognosis, provided of course that *RXFP4* was sufficiently expressed.

For the reasons stated above, a stable overexpression system for INSL5 in CRC cell lines was constructed, which enabled the finding that an overexpression of INSL5 can inhibit the proliferation of CRC cells (Fig. 6). The analysis of apoptosis pathway proteins revealed that overexpression of INSL5 can promote the cleavage of poly (ADP-ribose)-polymerase (PARP) (Fig. 6h, i). PARPs are a superfamily of multi-protein structures, which play an important role in DNA replication and DNA repair damage. Parthanatos is a cell death pathway that is not mediated by caspase [26]. It relies on PARP and mitochondrial-associated apoptosis-inducing factor (AIF). AIF nuclear translocation due to overactivation of PARP leads to large-scale DNA fragmentation and chromatin condensation, which leads to cell death and cell cycle arrest [27, 28]. This may be the related regulatory mechanism through which INSL5 affects progression of colorectal cancer, further research may be warranted to understand the precise mechanism. Based upon these results, inhibition of INSL5 (or activation of RXFP4 with specific agonist) could be explored as a potential strategy for CRC treatment.

HCAR3 is a metabolite-sensing GPCR [29]. Consistent with gene profiling data, *HCAR3* expression was higher in most CRC patient tissues, but the WB and IHC results showed no difference between tumor and its related adjacent tissues (Fig. 3). Moreover, no differences were observed among the CRC tissues. Kaplan–Meier survival curves showed that high expression of *HCAR3* was significantly associated with better overall survival of CRC patients (Fig. 3b). Therefore, it is possible that the biological functions of *HCAR3* might not be critical during CRC pathogenesis and progression. However, *HCAR3* might be a potential biomarker for CRC prognosis. To test this hypothesis, the prognosis of CRC patients was stratified based upon the expression of *INSL5* and *HCAR3*, in all but one case the prognosis was consistent with the expression of *INSL5* and *HCAR3* (Figure S2). This result indicated that *HCAR3* could be used as a biomarker for CRC prognosis. However, further studies are needed to illuminate its roles in CRC tumorigenesis.

It is noteworthy that this study has some limitations, which can be addressed in future studies by expanding upon the limited tumor sample size, with additional samples to verify the results. Finally, more in vitro experiments and the addition of in vivo experiments could help enhance our understanding of the key functions of these genes in the context of CRC.

## Conclusions

This integrated bioinformatics study presented 10 key hub genes associated with CRC, of which two were novel core genes. *HCAR3* and *INSL5* were expressed in tumor tissue and these were associated with poor survival and warrant further study with regard to their potential utility as CRC therapeutic targets.

## Supplementary Information

**Additional file 1: Table S1**. RT-PCR Primer Sets [30–32].

**Additional file 2: Figure S1** (a) Interaction networks for competing endogenous RNAs were produced as per the description in the supplementary information. The red rectangles indicate miRNA, green diamonds indicate lncRNA and purple circles represent mRNA. (b) UpSetR plot showing distribution of KEGG enrichment pathways for different genes. “Viral protein interaction with cytokine and cytokine receptor”; “Rheumatoid arthritis”; “IL-17 signaling pathway”; “Bile secretion”; “Cytokine-cytokine receptor interaction”.

**Additional file 3: Figure S2** Kaplan–Meier survival curve of INSL5/HCAR3 expression in colorectal cancer patients.

## Data Availability

All data generated or analyzed during this study are included in this published article [and its supplementary information files].

## Abbreviations

CRC: Colorectal cancer
DEGs: Differentially expressed genes
HCAR3: Hydroxycarboxylic acid receptor 3
INSL5: Insulin like 5
GEO: Gene Expression Omnibus
GO: Gene Ontology
KEGG: Kyoto Encyclopedia of Genes and Genomes
PPI: Protein–protein interaction
TCGA: The Cancer Genome Atlas
NPC: Nasopharyngeal carcinoma
RXFR4: Relaxin family peptide/ INSL5 receptor 4
GPCR: G protein-coupled receptor
PARP: Poly (ADP-ribose)-polymerase
AIF: Apoptosis-inducing factor
