# Supplementary material for: Identification and verification of HCAR3 and INSL5 as new potential therapeutic targets of colorectal cancer
Source: World J Surg Oncol. 2021 Aug 21;19:248. doi: 10.1186/s12957-021-02335-x (PMC8380340; doi:10.1186/s12957-021-02335-x)
Supplement: Supplementary file 1 — Additional file 1: Table S1. RT-PCR Primer Sets [30–32]. [file 12957_2021_2335_MOESM1_ESM.docx]

The mRNA network analysis

Identification of validated (miRecords, miRTarBase, TarBase databases) and predicted (DIANA-microT, ElMMo, MicroCosm, miRanda, miRDB, PicTar, PITA, TargetScan databases) interactions between selected miRNAs and hub genes was performed using the multiMiR package 1.2.0[[30](#_ENREF_30)] according to the reference manual. The starBase web server was used to decipher lncRNA-protein interactions. The obtained interactions were presented in the regulatory network and visualized using Cytoscape software [[31](#_ENREF_31)] (Figure S1a).

Crosstalk analysis

UpSetR [[32](#_ENREF_32)] was used to identify and plot the intersection between pathways (Fig. S1b), the individual hits correspond to their respective function, along with intersection sets from KEGG pathways.

**Table S1**: RT-PCR Primer Sets.

| **Gene** | **Primer Sets：5’→3’** |
| --- | --- |
| *𝛽-actin* (*ACTB*) | Forward primer: 5’-GTGGACATCCGCAAAGAC-3’ |
|  | Reverse primer: 5’-AAAGGGTGTAACGCAACTA-3’ |
| *HCAR3* | Forward primer: 5’-GCGTTCAGACTGGAAGTTTGG-3’ |
|  | Reverse primer: 5’-TCGTGCCACCG GAAGGTAT-3’ |
| *INSL5* | Forward primer: 5’- TCCAGCTCCCACATAAACGTG-3’ |
|  | Reverse primer: 5’-GCCATCAGTGCAACACAAAGTT-3’ |
