# Supplementary figures and images for: Identification and verification of HCAR3 and INSL5 as new potential therapeutic targets of colorectal cancer
Source: World J Surg Oncol. 2021 Aug 21;19:248. doi: 10.1186/s12957-021-02335-x (PMC8380340; doi:10.1186/s12957-021-02335-x)

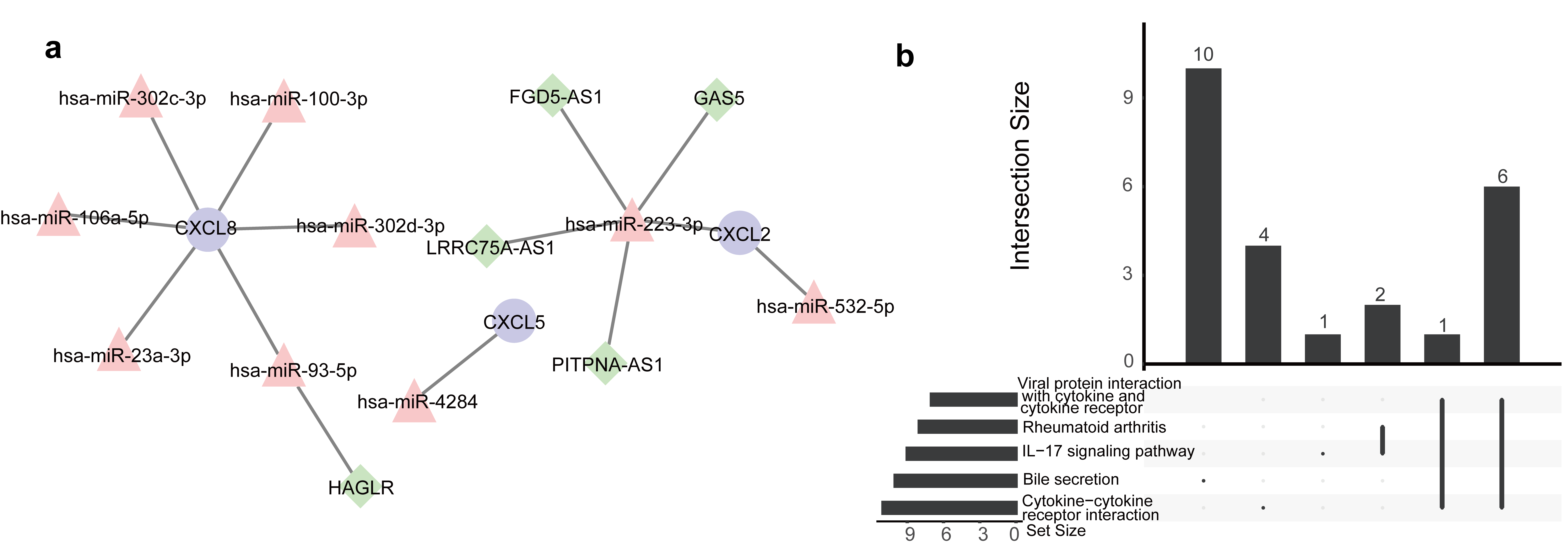

Supplement: Supplementary file 2 — Additional file 2: Figure S1 (a) Interaction networks for competing endogenous RNAs were produced as per the description in the supplementary information. The red rectangles indicate miRNA, green diamonds indicate lncRNA and purple circles represent mRNA. (b) UpSetR plot showing distribution of KEGG enrichment pathways for different genes. “Viral protein interaction with cytokine and cytokine receptor”; “Rheumatoid arthritis”; “IL-17 signaling pathway”; “Bile secretion”; “Cytokine-cytokine receptor interaction”. [file 12957_2021_2335_MOESM2_ESM.tif]

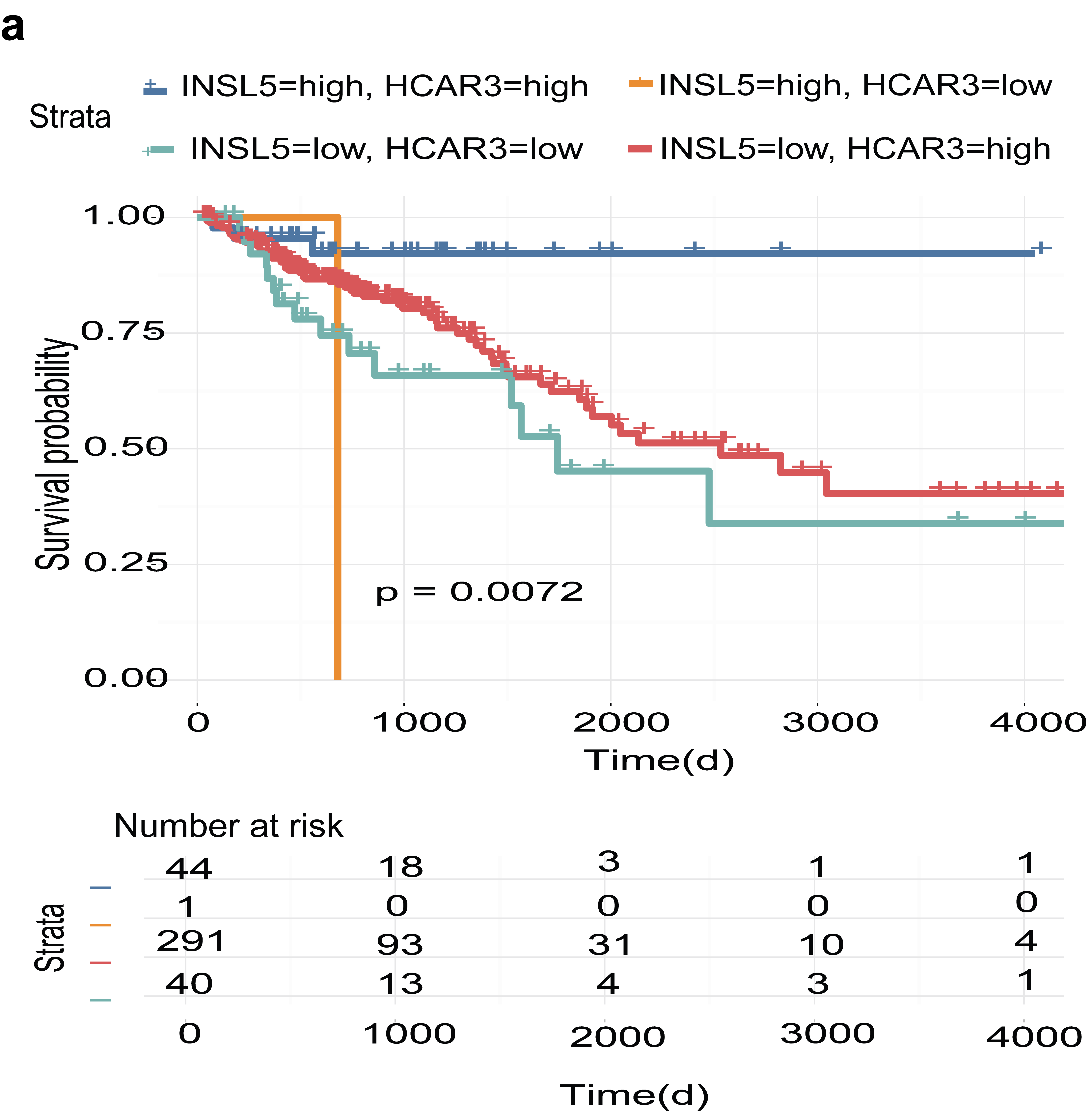

Supplement: Supplementary file 3 — Additional file 3: Figure S2 Kaplan–Meier survival curve of INSL5/HCAR3 expression in colorectal cancer patients. [file 12957_2021_2335_MOESM3_ESM.tif]
